# Combination of the endoscopic septonasal flap technique and bioabsorbable steroid-eluting stents for repair of congenital choanal atresia in neonates and infants: a retrospective study

**DOI:** 10.1186/s40463-021-00535-9

**Published:** 2021-08-12

**Authors:** Peng-peng Wang, Li-xing Tang, Jie Zhang, Xiao-jian Yang, Wei Zhang, Yang Han, Xiao Xiao, Xin Ni, Wen-tong Ge

**Affiliations:** 1 Department of Otorhinolaryngology Head and Neck Surgery, National Center for Children’s Health, Beijing Children’s Hospital, Capital Medical University, 56 Nan Li Shi Road Xi Cheng District, Beijing, 100045 People’s Republic of China; 2 Beijing Key Laboratory for Pediatric Diseases of Otolaryngology Head and Neck Surgery, Beijing Pediatric Research Institute, Beijing, 100045 People’s Republic of China; 3 Key Laboratory of Major Diseases in Children, Ministry of Education, Beijing, People’s Republic of China

**Keywords:** Choanal atresia, Bioabsorbable drug-eluting stent, Endoscopy, Infant, Neonate, Flap technique

## Abstract

**Background:**

Multiple surgical approaches have been proposed to repair the congenital choanal atresia. However, there remains no general consensus about the optimal surgical technique. This study aimed to describe and evaluate outcomes of the endoscopic septonasal flap technique combined with bioabsorbable steroid-eluting stents for repair of congenital choanal atresia in neonates and infants.

**Methods:**

Clinical data of 37 neonates and infants with congenital choanal atresia who received nasal endoscopic surgery with the flap technique between January 2018 and July 2020 were analyzed retrospectively. All patients underwent the ultra‑low‑dose paranasal sinus computed tomography imaging preoperatively to confirm diagnosis and plan the surgery. In these patients, the mirrored L-shaped flap technique was performed for bilateral atresia and the cross-over L-shaped flap technique was performed for unilateral atresia. A total of 22 patients had silicone stents postoperatively and 15 patients had bioabsorbable steroid-eluting stents postoperatively. Silicone stents were removed at one month postoperatively under secondary general anesthesia, while no anesthesia was needed to remove the bioabsorbable steroid-eluting stents. Postoperative follow-up ranged from 10 months to 3 years.

**Results:**

The septonasal flap technique was performed in all patients. Compared with the silicone stents group, the average operative duration and the hospital length of stay in the bioabsorbable steroid-eluting stents group were decreased [(97.46 ± 15.37) min vs (83.49 ± 19.16) min t = 13.733, *P* < 0.001] [(12.8 ± 3.22) d vs (7.67 ± 3.91) d t = 15.082, *P* < 0.001], the average number of procedures was reduced [(2.04 ± 0.64) vs (1.00 ± 0.001), t = 82.689, *P* < 0.001], the differences were statistically significant. There were no reports of postoperative restenosis and complications in the bioabsorbable steroid-eluting stents group, and follow-up endoscopic examinations showed patency and stable nasal passages in all cases.

**Conclusions:**

The endoscopic septonasal flap technique can effectively expose and expand the choanal bony structure for repair of congenital choanal atresia in neonates and infants. The combined use of this technique along with bioabsorbable steroid-eluting stents can help prevent the need for revision procedures and also against stent-related injuries.

## Background

Congenital choanal atresia (CCA) is the obliteration or blockage of the posterior nasal aperture that occurs in approximately 1 in 5000–8000 births [1]. It is one of the most common congenital defects and results in airway obstruction in neonates and infants [2]. The nature of the obstructing atretic plate has been described as 90% bony and 10% membranous in earlier studies. However, more recent studies have revealed that 30% of such malformations consist of a purely bony obstruction and 70% are a mixed bony-membranous obstruction [3]. Neonates are obligate nasal breathers, and bilateral CCA becomes apparent with the occurrence of acute respiratory distress and cyanosis [4].

Surgery is the definitive treatment for CCA and several approaches have been proposed to repair CCA. Within the past 30 years, the transnasal endoscopic approach has been preferred to repair CCA because it deals directly with the surgical field and allows removal of the atretic plate and posterior parts of the vomer to enlarge the choana [5–8]. Primary repair success rates reportedly range from 67 to 88% [9]. Postoperative restenosis remains a common complication of endoscopic CCA repair. To prevent restenosis and subsequent reoperation, many investigators advocate for preservation of the mucosa for use as flaps which in most cases is combined with postoperative stenting [10]. However, in recent years, stent-assisted repair has been controversial because stent-related injuries have been reported [11]. In 2016 and 2019, two studies reported using novel bioabsorbable steroid-eluting stents to treat CCA in 6 pediatric patients, and found it to be safe and effective with no restenosis in any patient [12, 13]. This study aimed to evaluate the outcomes of endoscopic repair of CCA that applied the septonasal flap technique with bioabsorbable steroid-eluting stents versus silicone stents.

## Patients and methods

### Study design and patient population

This is a retrospective study of patient data from 37 cases of congenital choanal atresia treated at Beijing Children’s Hospital from January 2018 and July 2020. Data of patients with pyriform aperture stenosis, congenital midnasal stenosis, severe facial deformities relate to midnasal stenosis (eg. Treacher-Collins, craniosynostosis), or postoperative death that unrelated to the surgery were excluded. Patient records were analyzed for age at surgery, sex, laterality, surgery technique, postoperative stenting, complications, and follow-up. Parents of all patients were fully informed about the method of treatment and provided signed informed consent for the therapy and later use of patient records. All patients were operated on by the first author as the primary surgeon. The study protocol was reviewed and approved by the board of medical ethics of Beijing Children’s Hospital.

**Fig. 1:**
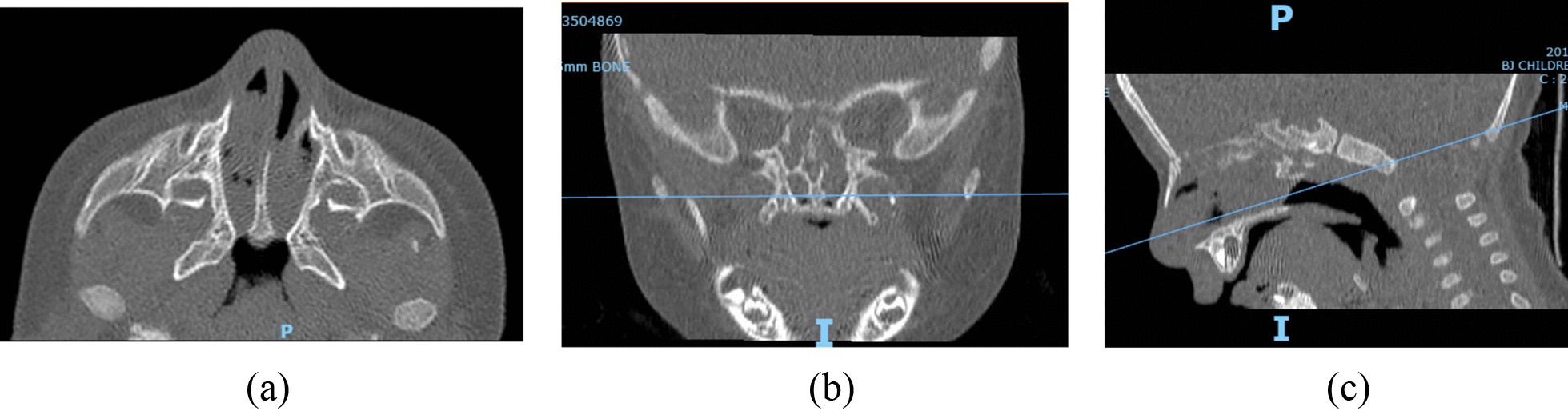
Reconstructed views of CCA nasal cavity. **a** Axial view. **b** Coronal view at level of pterygoid plates and **c** Sagittal view parallel to posterior hard palate

All patients underwent ultra-low-dose paranasal sinus computed tomography (CT) in layers of 0.625 mm in thickness using a 64-detector row CT scanner (Discovery CT750 HD, GE Healthcare, Chicago, IL, USA) with patients in the supine position [14]. The obtained axial image from CT was transferred to a workstation for analysis. The axial scanning section was parallel to the posterior hard palate at the level of the pterygoid plates, as described previously (Fig. 1a–c) [15]. Reconstructed views helped to indicate the safety margin of reference from along the floor of the nose to the nasopharynx preoperatively. It was essential to know preoperatively that surgery was possible without inadvertently dissecting too far superiorly towards the clivus or the sphenopalatine artery.

### Operative procedures

A HOPKINS II 0°, 2.7 mm endoscope (Karl Storz, Tuttlingen, Germany) was used under general anesthesia. The same surgical principles were followed in all patients: (1) application of adrenaline-soaked (1:10,000) pledgets to the nasal cavity for topical decongestion; (2) infiltration of the septum, atretic plates, and sphenopalatine foramen area with a mixture of 1% lidocaine and adrenaline (1:100,000 solution) using a 1-mm syringe and a number six spinal needle (0.5 ml per side); (3) incision and elevation of the mucosal flap to expose the surface of the thickened vomer and the lateral bone with a COTTLE elevator (Karl Storz) or a long needle-shaped electric knife (power output 15–20 w); (4) the thickened vomer and parts of the lateral bone were removed using pediatric backbiting forceps and a Medtronic diamond burr (Medtronic ENT, Medtronic, Minneapolis, MN, USA); and (5) at the end of the surgical procedure, stents were placed to keep the flaps in position and to maintain patency of the neo-choana.

### Septonasal flap techniques

The designs of the flap technique were based on our experience and inspired by the previous studies, adapted to the different types of laterality.

#### The mirrored L-shaped flap technique was designed for bilateral atresia

**Fig. 2:**
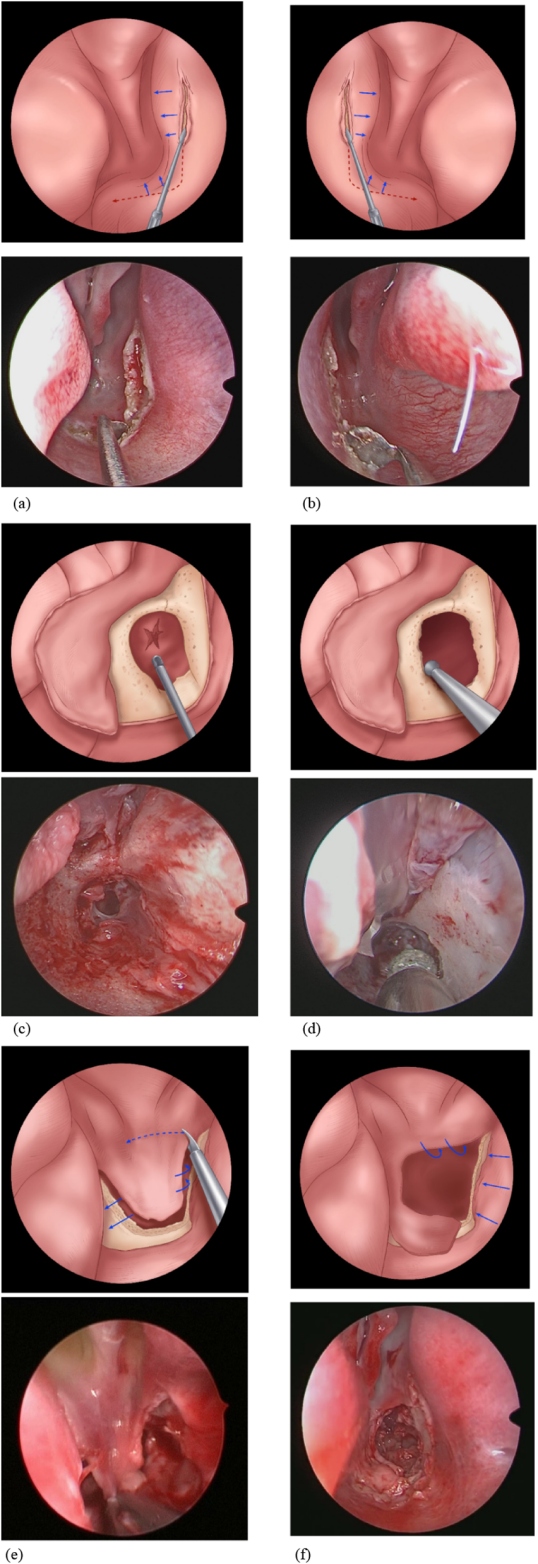
Mirrored L-shaped flap technique was designed for bilateral atresia (Each step is shown by Schematic diagram combined with real Endoscopic view). **a** Endoscopic view of the right nasal cavity showing the choanal atresia. **b** Endoscopic view of the left nasal cavity showing the choanal atresia. The red dashed line marks the incision made at the septal continue to nasal floor, reaching the lateral nasal wall. The long blue arrows mark the distance from the atretic plate to the septum incision, about 5–10 mm. **c** Elevation of the L-shaped septonasal flap and remove part of the pharyngeal face of the atresia plates by using a drill. **d** The thickened vomer and parts of the lateral bone were removed, widen the newly formed choana. **e** The septo-atretic flaps were fashioned by using the ear scissors. **f** The newly formed choana with the flap covering the lateral and inferior raw surface

A vertical incision was made from top to bottom at the cartilaginous/bony junction through the septal mucosa approximately 5–10 mm anterior to the atretic plate (the inferior aspect of the middle turbinate was used as a landmark for the superior limit of dissection, that can reduce the risk if cutting near or through the posterior septal artery), following to the floor of the nasal cavity, reaching the lateral wall of the nasal cavity (hence the name “L-shaped”) [3]. The mucosal flap over the thickened vomer and the nasal face of the atretic plate was elevated carefully to achieve maximal preservation of the mucosa and harvested flap. The same was done on the other side, creating a mirrored L-shaped septonasal flap (Fig. 2a, b).

After removing part of the pharyngeal face of the atresia plates using a Medtronic drill with a 2.9 mm blade (0 Silver bullet; Medtronic) (Fig. 2c), the thickened vomer and part of the lateral bone were removed using pediatric backbiting forceps and a Medtronic diamond burr (Medtronic ENT) (Fig. 2d). This step unified the neochoana in the midline. Once the adequate patency of the neochoana was enlarged to 6–10 mm in each side (at least 6 mm in neonates and at least 10 mm in patients over 1 year old), the preserved mucosal flap was fashioned (Fig. 2e) and folded over the lateral and floor raw bony areas and fixed with the stent (Fig. 2f).

#### The cross-over L-shaped flap technique for unilateral atresia

**Fig. 3:**
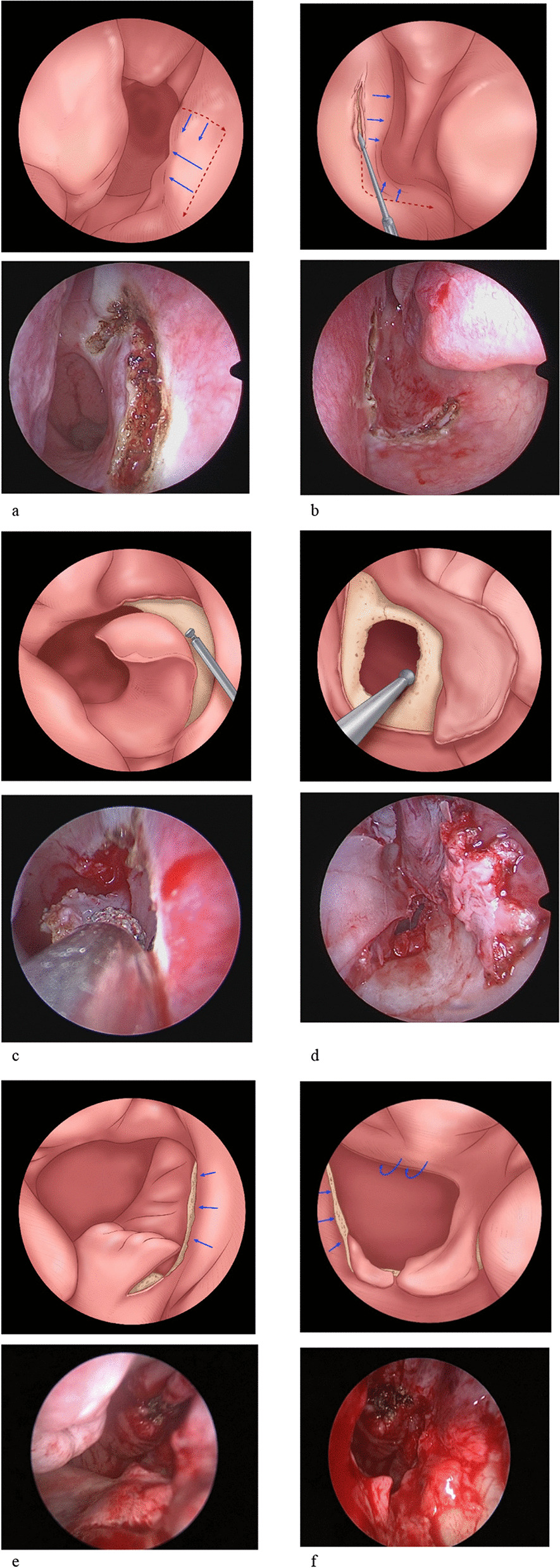
Cross-over L-shaped flap for unilateral atresia (Each step is shown by Schematic diagram combined with real Endoscopic view). **a** Endoscopic view of the healthy cavity showing the horizontal incision on the top of the septum from posterior to anterior, about 5–10 mm in length, and the vertical incision on the septum connected to the horizontal incision. **b** Endoscopic view of the left nasal cavity showing the choanal atresia. The red dashed line marks the incision made at the septal continue to nasal floor, reaching the lateral nasal wall. **c** After elevation of healthy side of the septal mucosal flap, thickened vomer in the healthy cavity was removed. **d** After elevation of the L-shaped septonasal flap in the atresia side, the pharyngeal face of the atresia plates and the thickened vomer and parts of the lateral bone were removed. **e** The healthy side mucosal flap rotated to cross the middle septal line to cover the floor and the border of the neo-choana. **f** The atresia side mucosal flap was fashioned and covering the lateral and inferior raw surface

An L-shaped incision was made in the atretic side, the same as done for the bilateral type (Fig. 3b). This mucosal flap should be preserved carefully because it provides the cover for the lateral wall and inferior posterior borders of the neochoana. In the healthy cavity, the mucosal flap was elevated at the posterior part of the septum using a horizontal incision at the top connected by the vertical incision approximately 5–10 mm anterior to the posterior septal edge (Fig. 3a), served as the flap that covered the floor and borders of the neochoana. This incision design was inspired by previous literature [16]. The bony plate and both sides of the thickened vomer were visualized and removed using a diamond drill or backbiting forcep (Fig. 3c, d). In the atretic side, patency of the new choana should be larger than the normal opening. The flaps were fashioned and folded over the raw bony areas and fixed with stent to keep the flaps in position and to maintain patency of the neochoana (Fig. 3e, f).

### Silicone stents and bioabsorbable steroid-eluting stents

**Fig. 4:**
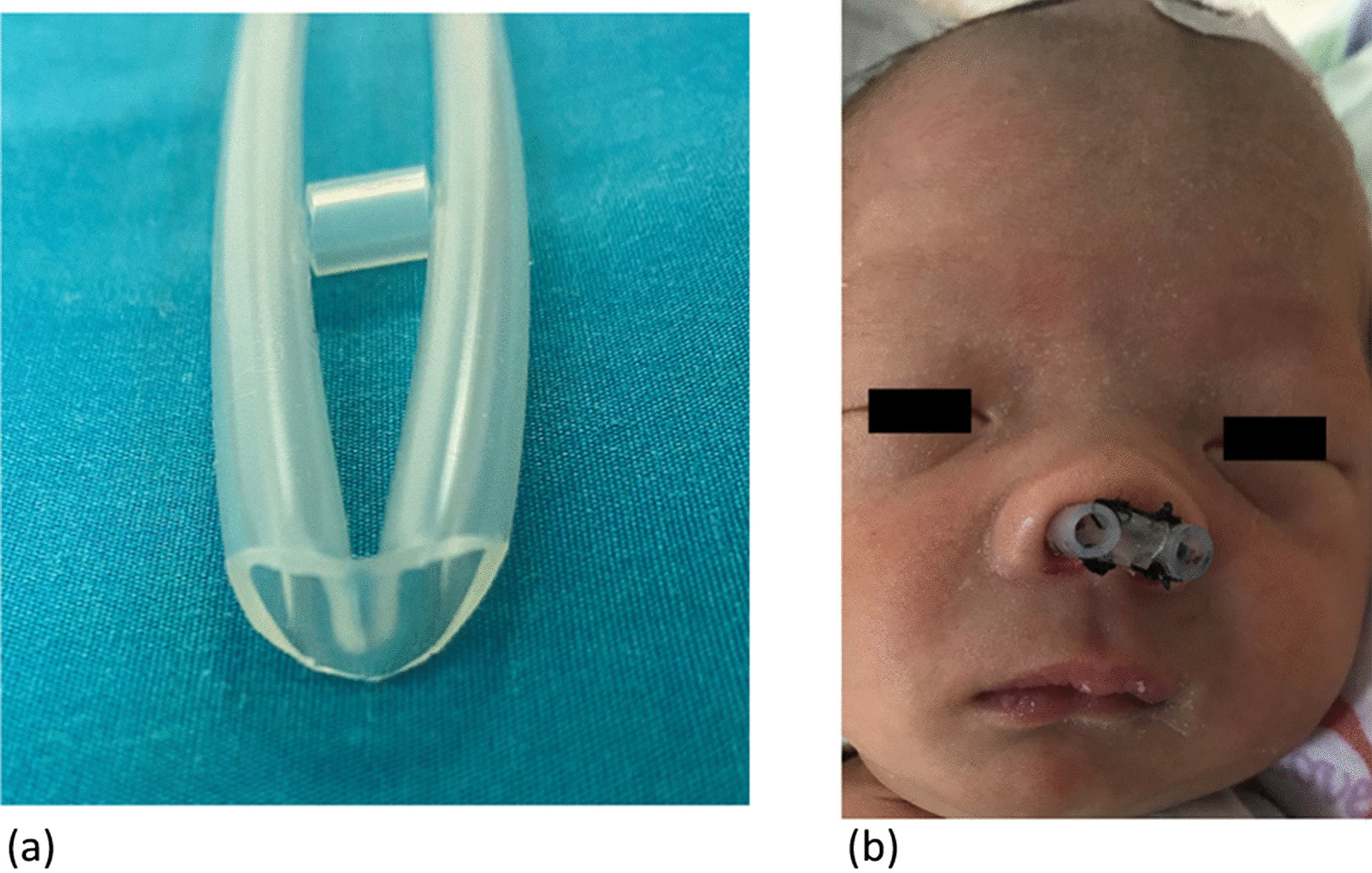
Customized silicone stents made by silicon suction catheter and how it placed. **a** The catheter was folded and a posterior fenestration was created, where dorsally a bridge was left behind which hinges around the vomer. **b** A small connecting piece was sutured between both nasal openings in order to prevent the postoperative displacement

**Fig. 5:**
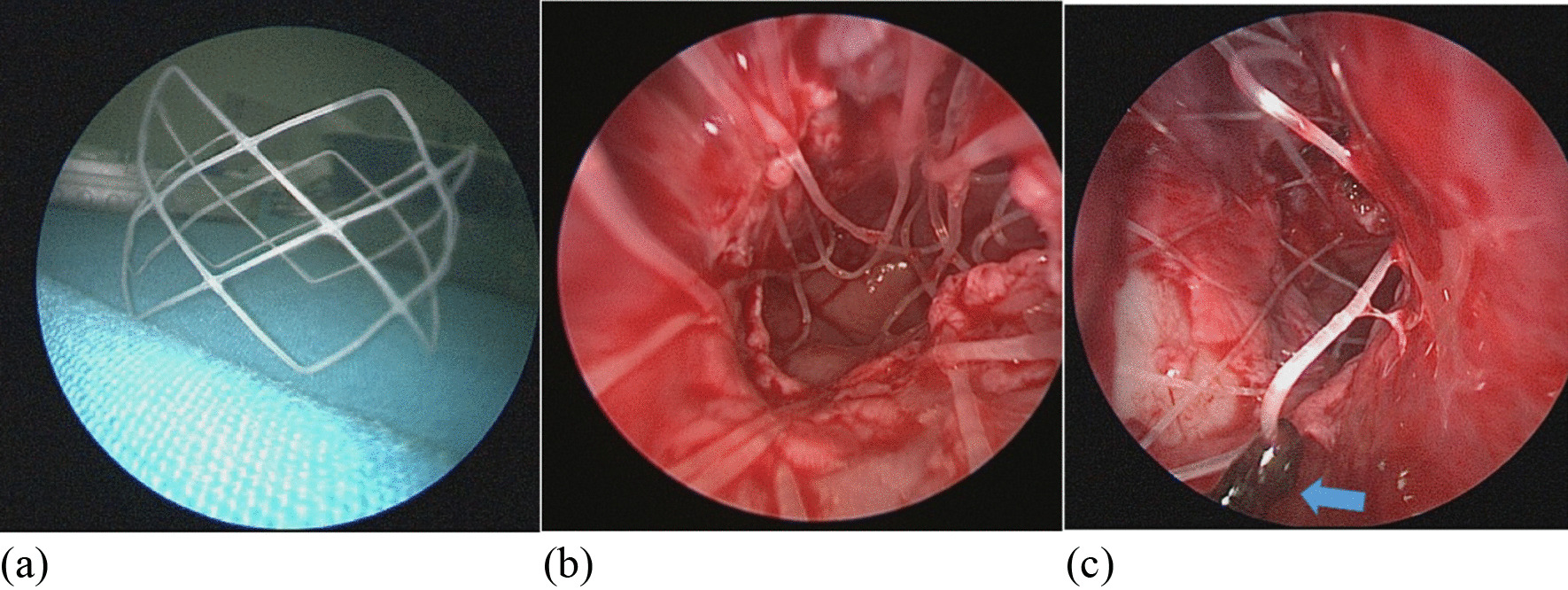
**a** The shape of bioabsorbable steroid-eluting stents. **b** Intraoperative placement of bioabsorbable steroid-eluting stents during primary repair of bilateral choanal atresia on a neonate. **c** A silk suture placed on the stent

Between January 2018 and June 2019, customized silicone stents (Fig. 4) were applied in 22 patients postoperatively [17]. Between July 2019 and July 2020, all 15 patients with CCA were implanted with bioabsorbable steroid-eluting stents (Fig. 5) (Xiangtong Sinus Stent, Puyi Biotechnology, Shanghai, China). The China Food and Drug Administration (CFDA) approved the Xiangtong sinus stent in November 2013 (ZL201210454911.2). The stent is composed of a bioabsorbable polylactide-co-glycolide polymer coated with 652 μg of a corticosteroid (mometasone furoate). The corticosteroid is released in a controlled fashion over approximately 30 days into the surrounding mucosa [18]. In our study, one silk suture was placed on the stent to avoid postoperative displacement of the stent.

### Postoperative care and assessment

Parents were informed about nasal irrigation and regular follow-up examinations. The silicone stent was kept for one month and removed gently via the mouth that usually under general anesthesia. The bioabsorbable steroid-eluting stent was designed to dissolve over 30 days of placement, so revision surgery was not needed for removal.

Postoperative assessments were performed one, three, and six months postoperatively and then one, two and three years postoperatively. Both subjective and objective assessments focused on the nasal obstruction symptoms and nasal endoscopy, respectively. Normal nasal patency was defined as a choanal aperture with < 50% restenosis using endoscopic examination and allowing normal airflow; partial restenosis was defined as anatomic stenosis > 50% and/or associated with symptoms (i.e., breathing, feeding, and growth difficulties); and complete stenosis was when no passage was visible under fibroscopy or endoscopy as previously described [19].

### Statistical analysis

Study data were analyzed using SAS version 9.4 statistical software (SAS Institute, Cary, NC, USA). Quantitative data were expressed as means ± standard deviations. Qualitative data were expressed as frequencies and percentages. The student t test of significance was used for comparisons of two means. The chi-square test of significance was used to compare proportions between two qualitative parameters. *P* values of less than 0.05 were considered statistically significant.

## Results

A total of 37 patients who underwent the septonasal flap technique were included in this retrospective study. The patient’s age at surgery ranged from 5 days to 3 years. Among all patients, 14 patients were male and 23 patients were female; 12 patients were neonates; 25 patients had associated malformations; 22 patients had bilateral atresia; and 15 patients had unilateral atresia. No isolated membranous obstruction was found in any patient. Of the total, 22 patients had silicone stents implanted and 15 patients had bioabsorbable steroid-eluting stents implanted. The groups were comparable in sex, surgical age, and laterality (*P* > 0.05) (Table 1).

**Table 1:** Clinical characteristics

Parameter	Silicone stents group (n)	Bioabsorbable steroid-eluting stents group (n)	Total (n)	Statistical test value	*P*-value
Sex				χ^2^ = 0.050	0.823
Male	8	6	14		
Female	14	9	23		
Age				χ^2^ = 0.947	0.731
0–1 month	7	5	12		
1–3 month	1	1	2		
3–6 month	5	2	7		
6–12 month	2	1	3		
1–3 year	7	6	13		
Tape of atresia				χ^2^ = 0.403	0.701
Mixed	21	15	36		
Bony	1	0	1		
Laterality				χ^2^ = 0.531	0.393
BCA	14	8	22		
UCA	8	7	15		
Associated malformations				χ^2^ = 0.417	0.659
Yes	16	9	25		
No	6	6	12		

χ^2^ = Chi-square test

No major intraoperative complications occurred in any case. The average operative duration and hospital length of stay in the bioabsorbable steroid-eluting stent group were shorter than the silicone stents group (Table 2).

**Table 2:** Postoperative data of Silicone stents Bioabsorbable steroid-eluting stents group

Parameter	Silicone stents group (n = 22)	Bioabsorbable steroid-eluting stents group (n = 15)	Statistical test value	P-value
Average operative duration (minutes)				
Mean ± SD	97.46 ± 15.37	83.49 ± 19.16	t = 13.733	0.000*
Number of procedures				
Mean ± SD	2.04 ± 0.64	1.00 ± 0.00	t = 82.689	0.000*
Hospital length of stay^▲^ (days)				
Mean ± SD	12.8 ± 3.22	7.67 ± 3.91	t = 15.082	0.000*
Restenosis (n(%))	2/22 (9.1%)	–	–	–
Timing of complications (days)	31.33 ± 5.12	–	–	–
Complications (n(%))	3/22(13.6%)	–	–	–
Granulations (n(%))	1/22(4.5%)	–	–	–
Damage of columella and alars (n(%))	1/22 (4.5%)	–	–	–
Posterior nasal septal injury (n(%))	1/22 (4.5%)	–	–	–

^*^ Indicates statistical significance between groups. SD = standard deviation; t = t-test

^▲^ Total length of hospitalization related to CA surgeries

**Fig. 6:**
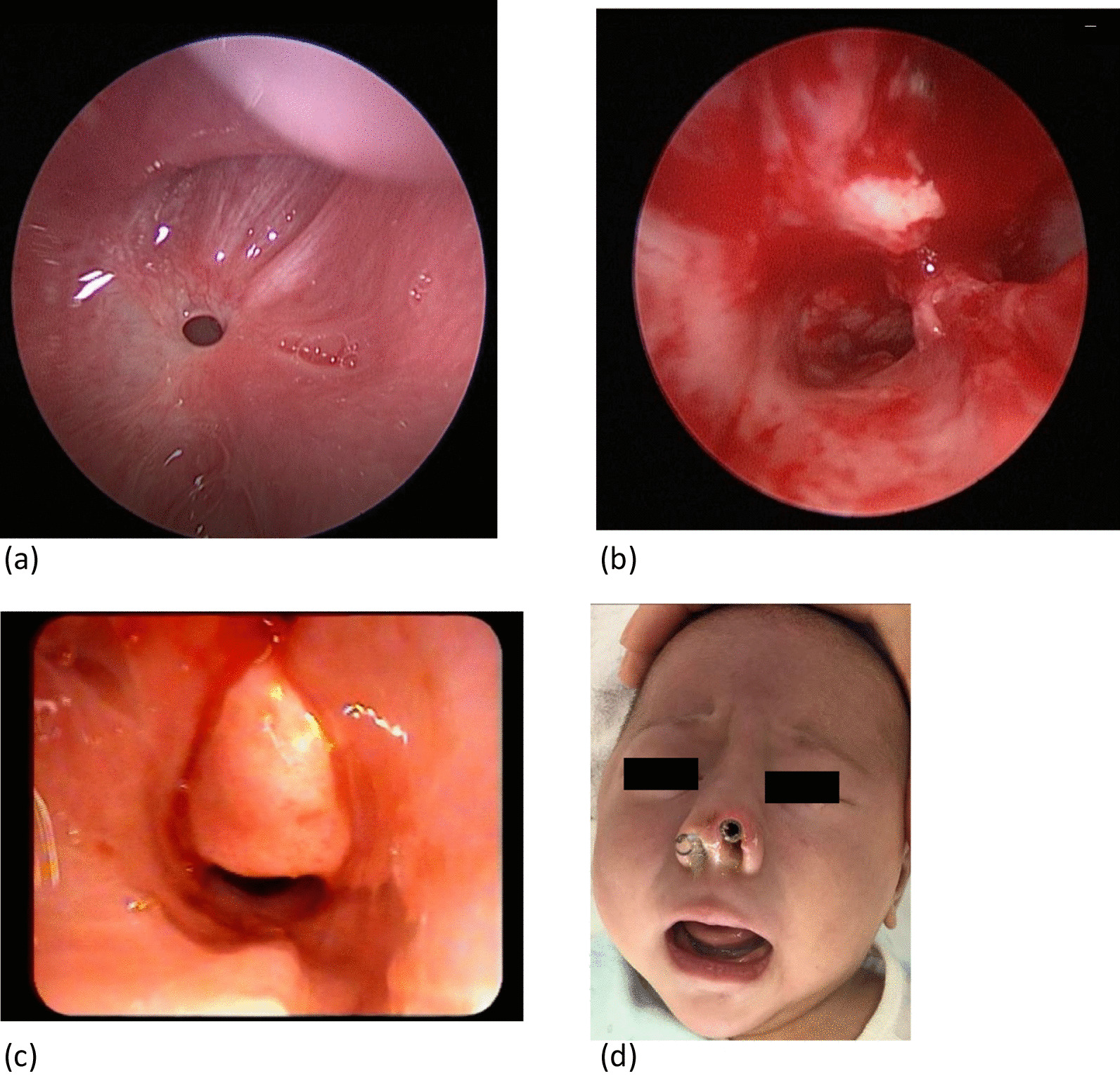
Silicone stents related complications. **a** Circumferential scar of the choanal aperture with > 50% restenosis using endoscopic examination. **b** Posterior nasal septal injury. **c** Granulation tissue present at time of stent removal. **d** The damage of columella and alars related to the silicone stent

The mean follow-up period for the silicone stents group was 27.7 ± 4.89 months (range of 21 to 36 months) after surgery and 14.6 ± 3.71 months (range of 10 to 21 months) after surgery for the group with bioabsorbable steroid-eluting stents. The silicone stents group had several postoperative complications including granulations, columella and alar damage, and posterior nasal septal injury (Fig. 6a–d). Complications were observed in 3 out of 22 patients (13.6%) at a mean of 31.33 ± 5.12 days (Table 2), and 2 out of 3 patients had the restenosis with the need for revision surgery was noted 1 and 3 mouths after stent removal respectively.

**Fig. 7:**
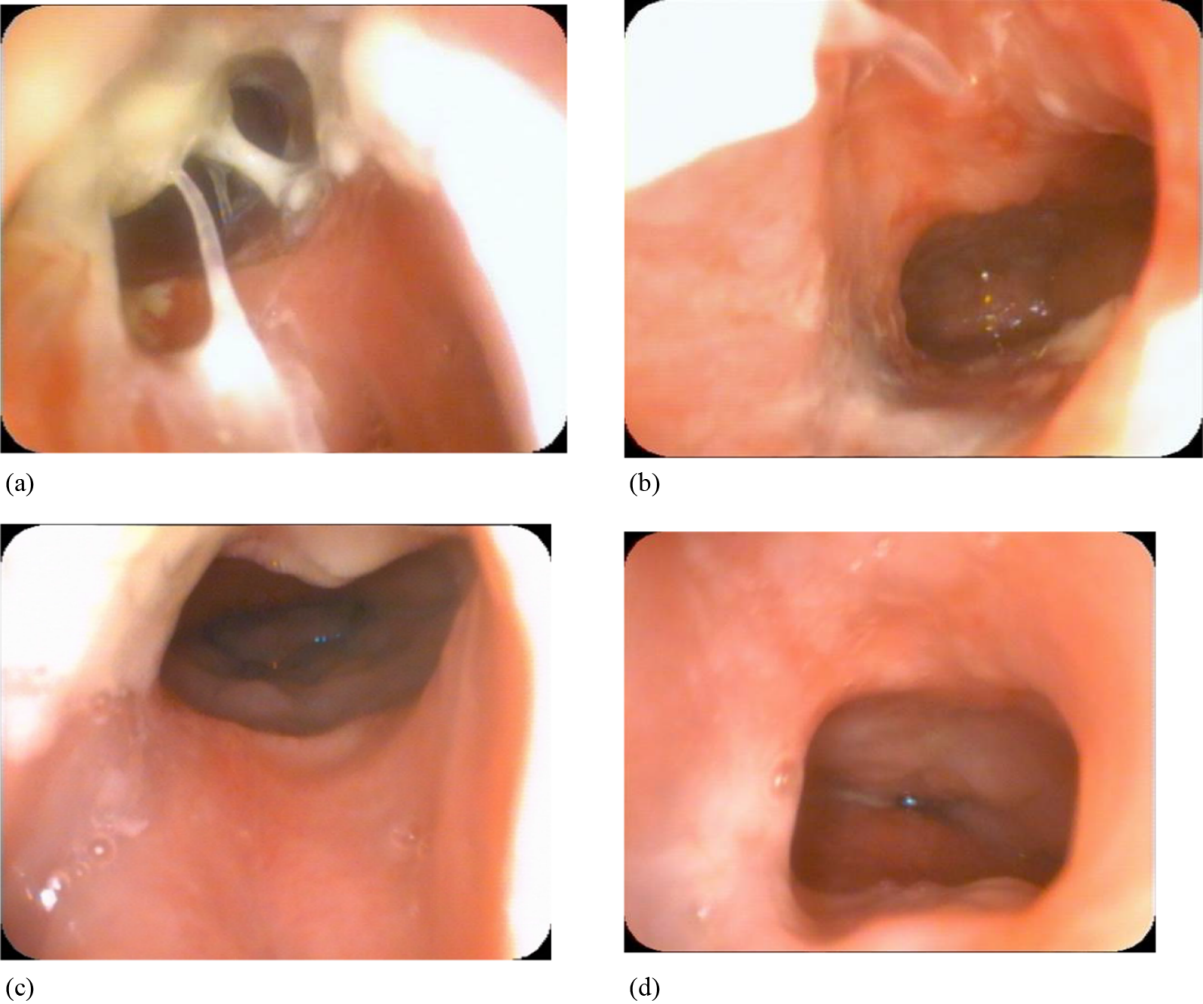
Post-operative endoscopic view of the choana after endoscopic repair of congenital choanal atresia using the septonasal flap technique combined the bioabsorbable steroid-eluting stents. **a** 2 weeks postoperatively; **b** 1 months postoperatively, the remnant bioabsorbable steroid-eluting stents almost dissolved. **c** 3 months postoperatively; **d** 6 months postoperatively

No postoperative complications were observed in the group with bioabsorbable steroid-eluting stents. Follow-up endoscopic examinations showed that the bioabsorbable steroid-eluting stents usually started to dissolve at the second week after the primary surgery. While it can still keep the nasal patency for 30 to 40 days until the stent dissolved totally (Fig. 7a–d), with a widely stable patent choana at more than 7–12 months in all cases. All 15 patients underwent only a single administration of general anesthesia at the time of the initial repair and did not require any additional revisions. At 10 to 21 months follow-up, all patients had patent nasal airways with no restenosis identified.

## Discussion

CCA was first described by Roederer in 1755 [9]. Since the use of endoscopic techniques for transnasal CCA repair was first demonstrated by Stankiewicz in 1990, it has become the primary procedure used by most surgeons [20]. Although different techniques and approaches for CCA correction have been extensively reported, a consensus on the recommended surgical techniques has not been reached because the majority of studies have been case series with a small sample size or consensus recommendations [4, 21]. Various studies have reported a range of cases between 12 and 54.7% that required a secondary surgical intervention, mainly because postoperative restenosis remains a common complication of endoscopic CCA repair [22]. Risk factors for restenosis include age, weight, bilateral choanal atresia [23], proper nasal patency was not achieved, and excessive growth of granulation tissue on the denuded bone [24]. It remains controversial how to reduce the incidence of postoperative restenosis in these patients. The two most common controversies regarding surgical repair of CCA are the use of stenting after surgery and the creation of flaps to cover the denuded bone.

The nature of the obstructing atretic plate has been was described as 90% bony and 10% membranous in 1910. With the advances of modern imaging techniques, 30% of such malformations consist of a purely bony obstruction and 70% are mixed bony-membranous obstructions with no purely membranous anomalies [9, 25]. Recent reports suggest that infants with bilateral choanal atresia have narrower posterior nasal skeletal borders than controls, while having a similar width of the pyriform aperture [26]. Two major osteological deformities have been described in choanal atresia: one is a medialization of the medial pterygoid plates and the other is a thickening of the posterior vomer [27]. Thus, we believe that for patients with CCA, resection of the thickened vomer, along with the careful removal of part of the lateral nasal wall, is crucial for maintaining long-term choanal patency.

Over the past three decades, drilling and mucosal debridement using a micro-debrider have become the gold standard in endoscopic approaches [23]. Recently, it has been suggested that the atretic plate and posterior parts of the vomer should be removed to expand the posterior choanal [7]. The lateral wall is the principle challenge of choanal atresia surgery, as most surgical corrections tend to address only the septum and atretic plate [28]. Certain advantages and disadvantages are attributed to each technique and there is still no unanimous agreement as to the ideal technique. Postoperative restenosis remains a common complication of endoscopic CCA repair. Restenosis may occur as a result of excessive growth of granulation tissue and fibrotic scar formation, especially associated with excessive drilling [2]. To avoid that possibility, covering as much of the row bone as poss ible with normal epithelium and preventing excessive trauma to the mucosa may help maintain the patency of a posterior CCA repair. For the purpose of preventing restenosis and subsequent reoperation, many authors advocate preserving the mucosa for use as flaps. From a 1990 report describing an initial pharyngeal mucosa flap with a star-shaped incision, variously designed shapes of mucosal flaps have been published, including the swinging door flap, mirrored L-shaped septonasal flap, nasal septal cross-over flap, and folded-over-flap [3, 8, 15, 23]. However, adopting the same mucosal flap design for different atretic types is impractical because of the limitations of each technique. In our study, we designed two different L-shaped incisions based on either unilateral or bilateral atresia: the mirrored L-shaped flap technique was performed in patients with bilateral atresia and the cross-over L-shaped flap technique was performed in patients with unilateral atresia. In patients with bilateral atresia, the laterally based mucosal flap was trimmed to co ver the neochoana boundary. In patients with unilateral atresia, the laterally based mucosal flap was designed for the atretic side, while the septum superior-based mucosal flap preserved the mucosa on the inferior choanal borders. This septonasal flap technique provided excellent visualization of the vomer and the lateral nasal bone to created adequate patency of the neochoana, and also the harvested flaps were used to resurface the postoperative raw bone surface. Accordingly, the entire circumference of the neochoana was covered by mucosa through a relatively simple technique.

Stents usually contribute to assisting the healing of mucosal flaps, avoiding early restenosis, and preventing post-surgery edema [29]. The positive effect of the duration of stenting on surgical outcomes was demonstrated in several studies, the recommended mean duration of stenting ranged from 7 days to 16 weeks11. Our center previous studies suggested stenting for at least 4–12 weeks, it can support the healing of mucosal flaps and allow patency until scarring has occurred to prevent restenosis [17]. However, stent-related disadvantages should be carefully considered. In our study, we had a few reports of silicone stent-related complications including the injuries and inflammation to surrounding tissue, where noted at average of 31.33 days after the primary surgery. Given the duration of stenting might be one of the factors for the complications, we had shortened the duration to 1 month, but the side-effects of using traditional stents were not completely avoidable. Meanwhile, the need for revision repair to the restenosis after stent removal, remained the other concern for the surgeon and patients. Recently, two studies describing the novel application of steroid-eluting stents have shown promising results in treating pediatric patients with CCA [12, 13]. This type of stent is composed of bioabsorbable polylactide-coglycolide, which is a polymer used in certain medical equipment, including surgical sutures [30]. It has a cylindrical fishnet shape and contains a spring-loaded mechanism, allowing it to conform to the shape of the cavity where it is placed [31]. The benefits of this type of stent include keeping the mucosal flaps in position and maintaining the patency of the neo-choana. Our study results demonstrated that the combination of the septonasal flap technique and the utilization of steroid-eluting stents at the site of atresia repair can not only can minimize trauma and complications, but also can achieve permanent functional patency in a one-step surgery. In previous studies particular to the pediatric population, there is a reported risk of sinus stents becoming gastrointestinal or airway foreign bodies [12]. To reduce this risk, we place one piece of silk suture on the stent, which has the dual benefit of preventing the stent from shifting into a foreign body and indicating the process of the stent's dissolution. Thus, we had no reports of complications or signs of aspiration in our case series.

A limitation of our study is that it was a retrospective review with a small sample size. In the future, we recommend enrollment of more patients randomized into groups to ensure a balance in sample size across groups over time. The second limitation in our study is the short follow-up period of the bioabsorbable steroid-eluting stents group. Therefore, the safety profiling and the long-term effects of this procedure in the pediatric population still needs further study.

## Conclusion

The endoscopic septonasal flap technique can expose and expand the choanal bony structure and prevent bone exposure in repair of CCA in neonates and infants. The use of bioabsorbable steroid-eluting stents solve the issue of postoperative complications caused previously by silicone stents, and they can effectively eliminate the need for revision procedures.

## Data Availability

The datasets used and/or analysed during the current study are available from the corresponding author on reasonable request.

## Abbreviations

CCA: Congenital choanal atresia
CT: Computed tomography
CFDA: China food and drug administration
