# Correction to: Combination of the endoscopic septonasal flap technique and bioabsorbable steroid-eluting stents for repair of congenital choanal atresia in neonates and infants: a retrospective study

**DOI:** 10.1186/s40463-021-00547-5

**Published:** 2021-11-02

**Authors:** Peng-peng Wang, Li-xing Tang, Jie Zhang, Xiao-jian Yang, Wei Zhang, Yang Han, Xiao Xiao, Xin Ni, Wen-tong Ge

**Affiliations:** 1 Department of Otorhinolaryngology Head and Neck Surgery, National Center for Children’s Health, Beijing Children’s Hospital, Capital Medical University, 56 Nan Li Shi Road Xi Cheng District, Beijing, 100045 People’s Republic of China; 2 Beijing Key Laboratory for Pediatric Diseases of Otolaryngology Head and Neck Surgery, Beijing Pediatric Research Institute, Beijing, 100045 People’s Republic of China; 3 Key Laboratory of Major Diseases in Children, Ministry of Education, Beijing, People’s Republic of China

## Correction to: Journal of Otolaryngology—Head & Neck Surgery (2021) 50:51 https://doi.org/10.1186/s40463-021-00535-9

Following publication of the original article [1], the authors would like to correct an error in the Abstract.

The affected sentence originally read:

Compared with the silicone stents group, the average operative duration and the hospital length of stay in the bioabsorbable steroid-eluting stents group were decreased [(97.46 ± 15.37) min vs (83.49 ± 19.16) min t = 13.733, *P* < 0.001] [(12.8 ± 3.22) d vs (7.67 ± 3.91) d t = 15.082, *P* < 0.001], the average number of procedures was reduced [(2.04 ± 0.64) vs (1.00 ± 0.001), t = 82.689, *P* < 0.001], the differences were statistically significant.

The sentence should read (the change has been highlighted in **bold typeface**):

Compared with the silicone stents group, the average operative duration and the hospital length of stay in the bioabsorbable steroid-eluting stents group were decreased [(97.46 ± 15.37) min vs (83.49 ± 19.16) min t = 13.733, *P* < 0.001] [(12.8 ± 3.22) d vs (7.67 ± 3.91) d t = 15.082, *P* < 0.001], the average number of procedures was reduced [(2.04 ± 0.64) vs (1.00 ± **0.00**), t = 82.689, *P* < 0.001], the differences were statistically significant.

The original article [1] has been corrected.
